# Dlg5 maintains apical polarity by promoting membrane localization of Crumbs during *Drosophila* oogenesis

**DOI:** 10.1038/srep26553

**Published:** 2016-05-23

**Authors:** Jun Luo, Heng Wang, Di Kang, Xuan Guo, Ping Wan, Dou Wang, Jiong Chen

**Affiliations:** 1 State Key Laboratory of Pharmaceutical Biotechnology and MOE Key Laboratory of Model Animals for Disease Study, Model Animal Research Center, Nanjing University, 12 Xue-fu Road, Nanjing, China 210061

## Abstract

Apical-basal polarity plays critical roles in the functions of epithelial tissues. However, the mechanisms of epithelial polarity establishment and maintenance remain to be fully elucidated. Here we show that the membrane-associated guanylate kinase (MAGUK) family protein Dlg5 is required for the maintenance of apical polarity of follicle epithelium during *Drosophila* oogenesis. Dlg5 localizes at the apical membrane and adherens junction (AJ) of follicle epithelium in early stage egg chambers. Specifically, we demonstrate that the major function of Dlg5 is to promote apical membrane localization of Crumbs, since overexpression of Crumbs but not other major apical or AJ components could rescue epithelial polarity defects resulted from loss of Dlg5. Furthermore, we performed a structure-function analysis of Dlg5 and found that the C-terminal PDZ3 and PDZ4 domains are required for all Dlg5’s functions as well as its ability to localize to apical membrane. The N-terminal coiled-coil motif could be individually targeted to the apical membrane, while the central linker region could be targeted to AJ. Lastly, the MAGUK core domains of PDZ4-SH3-GUK could be individually targeted to apical, AJ and basolateral membranes.

How cell polarity is established and maintained is an important question in the fields of cell and developmental biology. During animal development, polarized cells such as epithelial cells maintain their apical-basal polarity despite undergoing dramatic shape changes and tissue remodeling during morphogenesis. Genetic screens done in developing *Drosophila* and *C. elegans* have uncovered a number of highly conserved apical and basolateral regulators essential for the establishment and maintenance of apical-basal polarity^1^,^2^,^3^,^4^. Specifically, the Par3/Par6/aPKC complex and the Crumbs (Crb)/Stardust (Sdt)/Patj complex are thought to define and maintain the identity of apical membrane, and the Discs-large (Dlg)/Lethal giant larvae (Lgl)/Scribble (Scrib) complex delineates the basolateral membrane. Finally, the junctional complex of E-cadherin/β-catenin/α-catenin initiates and maintains the adherens junction (AJ), which divides the cortex into apical and basolateral regions. Decades of research have formed a consensus model, in which the apical-basal polarity is generated and maintained by a mutually antagonistic interaction between the apical regulators and the basolateral regulators^5^,^6^,^7^. Recently, a mathematic modeling study done in *Drosophila* follicle epithelia suggested that in addition to the negative feedback between apical regulators and basolateral regulators, a positive feedback loop among apical polarity regulators is required to maintain the apical-basal polarity in epithelia^8^. A central component of this positive feedback loop is the transmembrane protein Crb, and its apical membrane localization was thought to be the key to maintenance of apical-basal polarity.

Discs-large 5 (Dlg5) belongs to the MAGUK family, and it is highly conserved across species including human, mouse, chicken, zebra fish and *Drosophila*^9^. MAGUK members also include Dlg and Sdt, which act as molecular scaffolds and are core components of the basolateral Dlg/Lgl/Scrib complex and the apical Crumb complex respectively. Dlg5 was first identified in human and was found to be expressed in placenta and in prostate gland epithelia^10^. Since then, Dlg5 was found to interact with a variety of junctional, cytoskeletal, trafficking, and receptor molecules, including β-catenin, P55, vinexin, Girdin, Citron kinase, Syntaxin, Smoothened and TGF-β receptors^9^,^10^,^11^,^12^,^13^,^14^,^15^. And its functions vary from inhibiting cancer cell migration to mediating receptor signaling, but most of these functions were obtained from cell culture studies. Detailed genetic analysis of *Dlg5* was first done using *Dlg5* knockout mice, which displayed failure of epithelial tube maintenance resulting in brain hydrocephalus and kidney cysts^9^. These defects were likely due to disruption of apical polarity and AJ. This study focused on Dlg5’s requirement of AJ function and found that Dlg5 physically interacted with the β-catenin/cadherin complex and was found together with β-catenin/cadherin complex in both the Rab11-labeled recycling vesicles and the AJ. A more recent work using the same *Dlg5*^−/−^ mice found that *Dlg5* was also required for lung morphogenesis^16^. Specifically, deletion of *Dlg5* resulted in loss of apical polarity markers such as aPKC, and Dlg5 was partially colocalized with aPKC in the apical membrane of the wild type lung epithelia^16^. But how Dlg5 exerts its functions in the apical membrane and regulate apical polarity is unknown.

In *Drosophila*, RNAi knockdown of *dlg5* affected the cohesion and morphology of border cell clusters as well as delaying their migration^17^. Recently, genetic analysis of *Drosophila Dlg5* revealed that its mutation caused embryonic lethality and loss of germ cells in the embryonic gonad^18^. Moreover, reduction of Dlg5 in the follicle cells in the adult ovary leads to defects in egg chamber budding, stalk cell overgrowth, ectopic polar cell induction and abnormal distribution of E-cadherin^18^. However, detailed analysis of whether and how *Drosophila* Dlg5 regulates epithelial or apical polarity has not been done. Here, we report that a genetic screen for follicle epithelial morphogenesis has identified the *Drosophila* Dlg5 as an essential player for maintenance of apical polarity by promoting Crb’s apical membrane localization.

## Results

### *dlg5* is required for follicle epithelial morphogenesis

**Figure 1:**
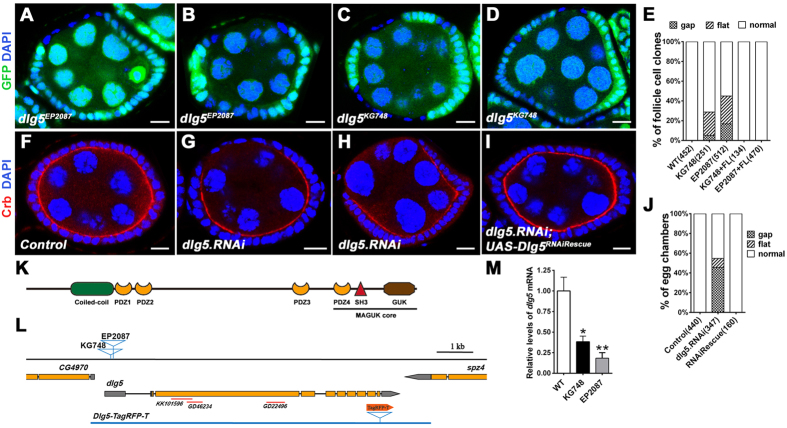
Dlg5 is required for follicle epithelial morphogenesis. (**A–D**) Mutant clones of *dlg5*^*EP2087*^ (**A,B**) and *dlg5*^*KG748*^ (**C,D**) displayed morphological defects in follicle epithelia including cell flattening (**A,C**) and gap (**B,D**). Mutant clones were marked by the absence of GFP (green), nuclei were stained with DAPI (blue). (**E**) Quantification of morphological defects as displayed in (**A**–**D**), as well as from those rescued by *dlg5* full length cDNA (number of clones indicated in parenthesis beside each genotype). (**F–I**) Knockdown of *dlg5* caused similar epithelial defects as in (**A**–**D**). Control (*act5C-GAL4,tub-GAL80*^*ts*^*/+*) egg chambers showed normal morphology of follicle cells (**F**). *act5C-Gal4,tub-Gal80*^*ts*^/*UAS-dlg5.RNAi* flies were temperature shifted to 29 °C for 3 days and the egg chambers displayed cell flattening (**G**) and gap (**H**) phenotype. These phenotypes were rescued by *UAS-Dlg5*^*RNAiRescue*^ (**I**). Ovaries were stained with Crb (red) and DAPI (blue). (**J**) Quantification of morphological defects as displayed in (**F–I**) (number of egg chambers indicated in parentheses). (**K**) The domain organization of *Drosophila* Dlg5 protein. (**L**) Gene structure and mutant alleles of *dlg5*. Orange boxes represent the coding sequences, and grey boxes represent untranslated regions. The red lines indicate the sequences used in the VDRC RNAi lines. (**M**) The mRNA levels of *dlg5* were reduced in *dlg5* mutants as compared with the wild-type flies. Experiments were repeated independently three times with consistent results and three replicates were used per time point. Error bars represent s.e.m.; *P = 0.0263, **P = 0.0087, Student’s *t* test. Scale bars: 10 μm.

We carried out a P-element based loss-of-function screen to identify new genes required in follicle epithelium morphogenesis during *Drosophila* oogenesis. Using genetic mosaic methods (FLP/FRT), we screened through a collection of FRT-P-element lethal mutations described previously^19^. We identified 2 P-element lines KG748 and EP2087 in which mosaic egg chambers containing homozygous mutant clones displayed strong morphogenesis defects in follicle epithelia (Fig. 1A–D). The gross morphological defects in the mosaic clones range from gaps between heterozygous tissues (severe) to various degrees of cell flattening (less severe). For EP2087 and KG748, 45% and 28% of follicle cell clones respectively showed morphology defects (Fig. 1E). Among the clones with defects, 38% in EP2087 and 18% in KG748 displayed the more severe gap phenotype, while 62% in EP2087 and 82% in KG748 exhibited the cell flattening phenotype (Fig. 1E). Both EP2087 and KG748 contained a single P-element insertion in the 5′ untranslated region of the gene *CG6509* (Fig. 1L), which encodes the *Drosophila* homolog of Dlg5, a MAGUK family member (Fig. 1K). Homozygous EP2087 and KG748 mutants died at 2^nd^ and 3^rd^ instar larval stage respectively, and the two P-element mutations failed to complement each other in terms of lethality. Real-time PCR data indicated that mRNA levels of *dlg5* in EP2087 and KG748 mutant larva showed 82% and 62% reduction respectively, as compared with the wild-type (Fig. 1M). Based on the degree of mRNA reduction, the lethality stage and the severity of follicle epithelial defects, we concluded that both were loss-of-function alleles of *dlg5*, with *dlg5*^*EP2087*^ being a stronger hypomorphic allele than *dlg5*^*KG748*^.

Lastly, expressing *dlg5 RNAi* in the adult ovary by *act5C-GAL4/tub-GAL80*^*ts*^ (temperature sensitive GAL80) also produced similar follicle epithelial defects with those of *dlg5*^*EP2087*^ and *dlg5*^*KG748*^, and the phenotype could be rescued by a rescue transgenic construct (Fig. 1F–J). To further determine whether the lethality and follicle defects of *dlg5*^*KG748*^ and *dlg5*^*EP2087*^were due to loss of *dlg5* expression, we performed genetic rescue experiments. Both a 23kb P[acman] BAC fragment that covered the full length of *dlg5* locus and a Flag-tagged *dlg5* cDNA construct expressed by *Ubi-63E* promoter were able to completely rescue the lethality and fertility of both *dlg5*^*EP2087*^ and *dlg5*^*KG748*^ (Supplementary Table S1). Moreover, follicle epithelial morphology of the rescued adult females was normal (data not shown), indicating that both alleles were bona fide *dlg5* mutations.

### Dlg5 deficiency specifically affected maintenance of apical polarity and AJ

**Figure 2:**
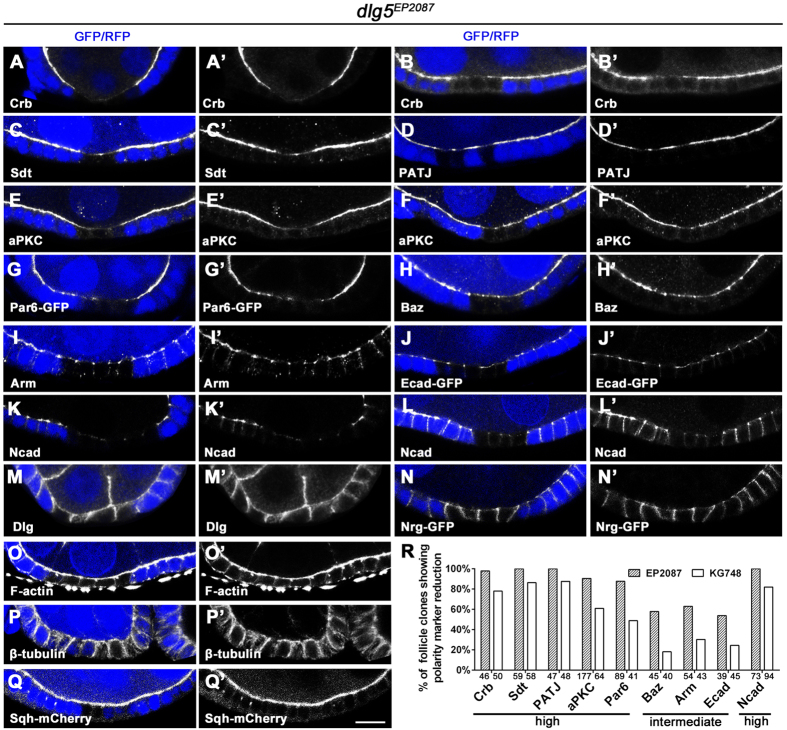
Dlg5 is specifically required for apical polarity and AJ. (**A–L’**) *dlg5*^*EP2087*^mutant clones in follicle epithelia displayed reduction of apical and AJ proteins including Crb (**A–B’**), Sdt (**C,C’**), PATJ (**D,D’**), aPKC (**E**–**F’**), Par6 (G,G’), Baz (**H,H’**), Arm (**I,I’**), E-cad (**J,J’**), N-cad (**K**–**L’**). The reduction of polarity proteins is stronger in clones with cell flattening (**A,A’,E,E’,K,K’**) than in clone with normal morphology (**B,B’,F,F’,L,L’**). (**M–Q’**) Basolateral and septate junction proteins Dlg (**M,M’**), Nrg (**N,N’**) and cytoskeletal components F-actin (**O,O’**), β-tubulin (**P,P’**), Sqh (**Q,Q’**) were not affected in *dlg5*^*EP2087*^ mutant clones. Mutant clones were marked by the loss of GFP or RFP (blue). (**R**) Quantification of the percentage of *dlg5*^*EP2087*^ or *dlg5*^*KG748*^ follicle cell clones that showed significant reduction of the polarity proteins (number of clones indicated below x-axis). The reduction of Crb, Sdt, PATJ, aPKC, Par6 and Ncad has high penetrance (>87% for *dlg5*^*EP2087*^) whereas the reduction of Baz, Arm and Ecad has intermediate penetrance (<63% for *dlg5*^*EP2087*^). Scale bar: 10 μm.

**Figure 3:**
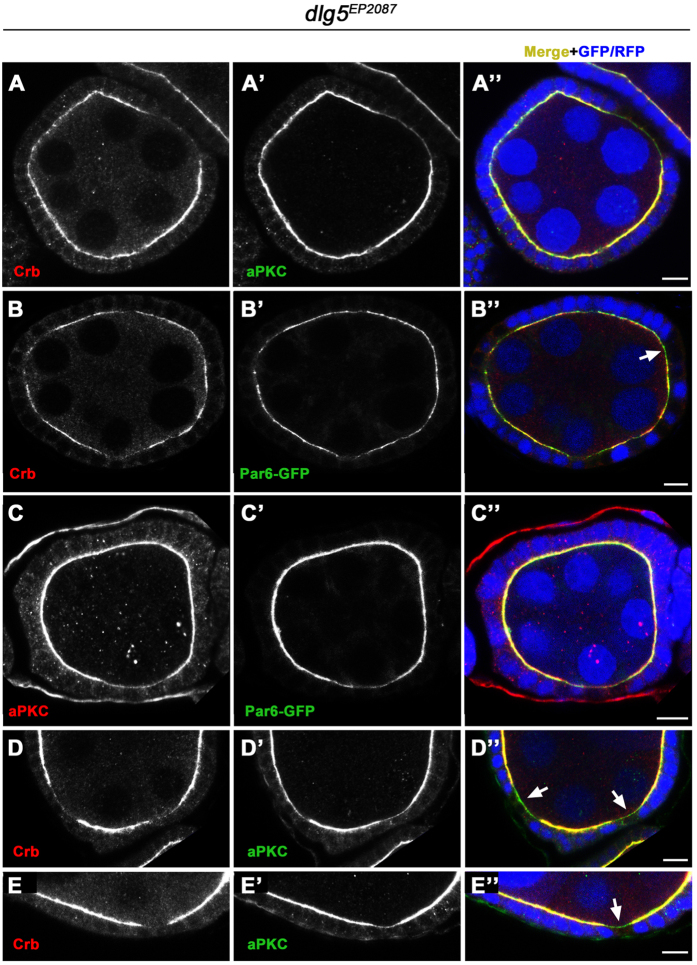
Double staining of apical proteins in *dlg5* mutant clones. (**A–C”**) In most cases, the degrees of reduction of Crb, aPKC and Par6 were comparable in *dlg5*^*EP2087*^ mutant clones. The arrow in B” indicates a site where Crb was almost lost but Par6 was still present. (**D–E”**) In a minority of *dlg5*^*EP2087*^ mutant clones, the reduction of Crb is distinctly stronger than that of aPKC. Arrows in (**D”,E”**) indicate the mutant clones where Crb was lost but aPKC was still present in a significant amount. Mutant clones were marked by the loss of GFP or RFP (blue). Scale bars: 10 μm.

The follicle epithelium defects suggested a loss of apical-basal polarity or cytoskeletal integrity. To determine the cause of these defects, we first examined the localization of apical complexes in the *dlg5*^*EP2087*^ mutant clones. The Crb complex components, Crb, Sdt and Patj each showed strong reduction in the apical membrane domain in *dlg5*^*EP2087*^ mutant clones as compared to the adjacent wild-type tissues (Fig. 2A–D’). Similarly, aPKC and Par6, two core components of the apical Par complex, also displayed strong reduction in the apical domain of the mutant epithelial cells (Fig. 2E–G’). Furthermore, co-immunostaining experiments demonstrated that the extent of reduction between Crb and aPKC, Crb and Par6, or aPKC and Par6 was mostly comparable (Fig. 3A–C”), except that in a small portion of *dlg5* mutant clones Crb’s reduction was more severe than that of aPKC or Par6 (Fig. 3D–E”). On the other hand, components of the sub-apical AJ including E-cadherin (E-cad) and Arm (β-catenin) displayed only moderate reduction (Fig. 2I–J’). It was previously reported that the AJs of the follicle epithelium contain both E-cad and N-cadherin (N-cad) up to stage 9 of oogenesis^20^,^21^, and we found that N-cad was more strongly reduced than E-cad and Arm in the mutant clones (Fig. 2K–L’). Interestingly, Baz (Par3), which is another component of Par complex and is distributed in both the apical membrane and sub-apical AJs (unlike aPKC and Par6), displayed only moderate reduction in both regions (Fig. 2H,H’). Furthermore, reduction in levels of these polarity components were observed both in clones with cell flattening defects and in clones with normal morphology, but mutant clones with the defects in general exhibited a more severe loss of these markers than mutant clones with normal appearance (Fig. 2A–B’’). This indicated that reduction or loss of apical and AJ molecules preceded cell flattening and morphological disruptions. Consistently, *dlg5 RNAi* resulted in a similar degree of reduction of various apical and AJ markers in egg chambers as early as stage 1 (Fig. S1). Importantly, after only 12 hours of *dlg5* RNAi, significant reduction of apical and AJ markers was observed in stage 6/7 and early stage 9 egg chambers (Fig. S1S–V’). These results indicate that Dlg5 is specifically required for the maintenance of apical polarity and AJ, since only 12 hours of Dlg5 deficiency would not have affected the establishment of apical polarity and AJ for the stage 6/7 or stage 9 follicle epithelia (for it takes more than 30 or 40 hours for follicle precursors in the germarium to develop into stage 7 or stage 9 follicle epithelia respectively^22^). Therefore, only the maintenance of apical polarity and AJ could be affected by the loss of Dlg5 within a 12-hour window.

We then quantified the percentage of *dlg5*^*EP2087*^ follicle cell clones that showed significant reduction of apical or sub-apical markers. Around 90% of *dlg5*^*EP2087*^ clones displayed Crb, Sdt, PATJ, aPKC, Par6 and N-cad reduction, indicating a high penetrance, whereas about 60% of *dlg5*^*EP2087*^ clones displayed significant reduction of Baz, Arm and E-cad (Fig. 2R). Interestingly, markers that generally displayed strong reduction have high penetrance, while markers that generally displayed moderate reduction have intermediate penetrance. Finally, mutant clones of the weaker *dlg*^*KG748*^ allele also displayed similar reduction of apical and AJ markers as compared with *dlg5*^*EP2087*^ (data not shown), but with an overall lower penetrance (Fig. 2R).

To further confirm that defects in apical polarity and AJ in *dlg5*^*EP2087*^ or *dlg5*^*KG748*^mutant clones were caused by loss of function of *dlg5*, we utilized two *dlg5* transgenes to perform phenotypic rescue experiment. The first one is a genomic construct *Dlg5-TagRFP-T* that contains a 9.6kb genomic sequence encompassing the entire *dlg5* genomic locus with a *RFP* tag sequence inserted in the C-terminus of *dlg5 ORF* (Fig. 1L). The second transgenic construct *Ubi-Dlg5.EGFP* allows ubiquitous expression of *dlg5-GFP* (C-terminal fusion of *GFP*) under the control of the *Ubi-63E* promoter. Both constructs were able to rescue the lethality of *dlg5*^*KG748*^, *dlg5*^*EP2087*^, *dlg5*^*KG748*^*/Df(2L)BSC242* and *dlg5*^*EP2087*^*/Df(2L)BSC242* (Table S1), indicating that both transgenes were functional. Importantly, *Dlg5-TagRFP-T* and *Ubi-Dlg5.EGFP* were able to fully rescue all the epithelial defects including loss of apical polarity and AJ markers as well as cell flattening and gaps in *dlg5*^*EP2087*^ mutant clones (Fig. S2 and S5F–G”).

We next determined whether the defects were specific only to apical and AJ regions. We found that basolateral and septate junctional markers, such as Dlg and Nrg respectively, appeared properly localized to their supposed positions (along the entire length of basolateral membrane) in *dlg5* mutant follicle cell clones (Fig. 2M–N’). Importantly, unlike apical and sub-apical markers, basolateral markers displayed no significant reduction in their levels in both morphologically normal and abnormal (flattened) follicle cells. Lastly, we observed no gross defects in cytoskeletal distribution in *dlg5* mutant clones, as indicated by stainings for F-actin, tubulin and Sqh (myosin light chain) (Fig. 2O–Q’). Taken together, these results demonstrated that Dlg5 is specifically required for apical polarity and AJ maintenance but not for basolateral polarity and septate junctions in follicle epithelia undergoing morphogenesis.

### Dlg5’s subcellular localization and expression patterns

**Figure 4:**
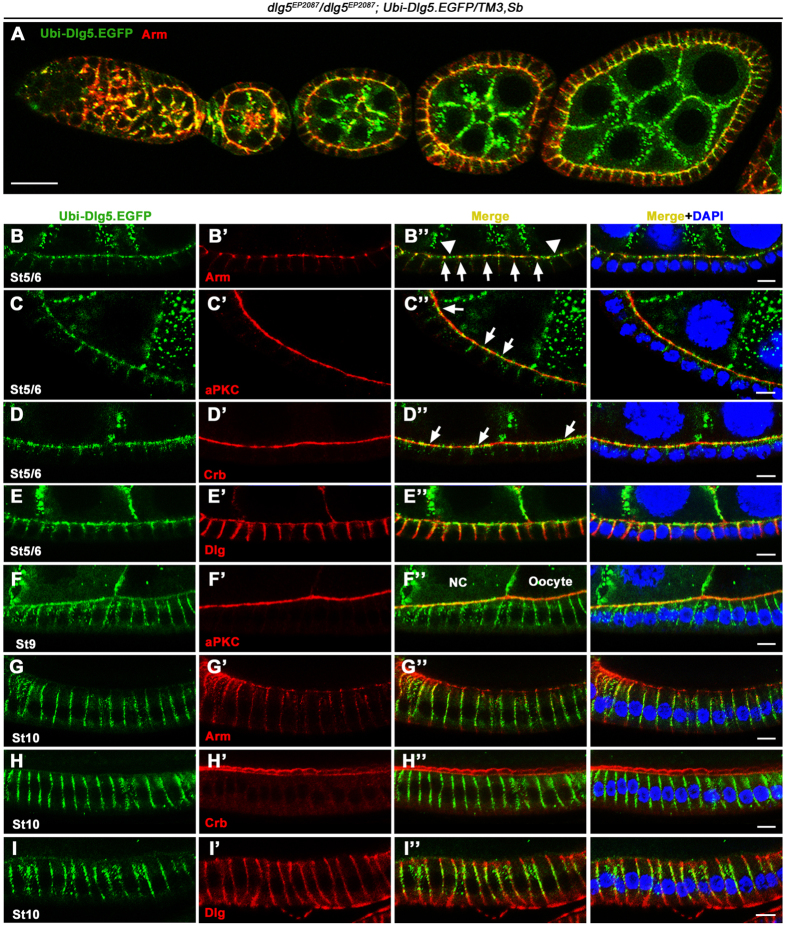
Dlg5 localizes to the apical domain and AJ in early stage egg chambers and to the basolateral domain in stage 10 egg chambers. (**A**) The confocal image of an ovariole shows that Dlg5-GFP colocalizes with Arm in germarium and early stage egg chambers. (**B**–**B”**) Dlg5-GFP (green) colocalized with Arm (red) at AJs in early stage egg chambers (indicated by arrows in **B”**). Dlg5-GFP spots also localized at apical domain between adjacent AJs (indicated by arrowheads in B” and correspond to spots in Fig. 6A–C’”). (**C–D”**) Dlg5-GFP spots localizes to the apical domain marked by aPKC (**C**–**C”**) or Crb (**D**–**D”**). Arrows indicate the Dlg5-GFP spots localized in the apical domain (compare to spots in Fig. 6A–C’”). (**E–E”**) In early stage egg chambers, Dlg5-GFP also localized to part of the lateral membrane marked by Dlg, but its colocalization with Dlg is restricted to regions adjacent to apical domain. (**F–F”**) In stage 9 egg chambers, Dlg5-GFP localized to both apical membrane and basolateral membrane. Note that the apical localization is stronger in follicle cells surrounding the nurse cells (NC) than that surrounding the oocyte. (**G–I”**) In stage 10 egg chambers, Dlg5-GFP localized to most of the basolateral domain marked by Dlg (red, **I–I”**), but did not localize to the AJ spots and apical membrane, marked by Arm (**G–G”**) and Crb (**H–H”**) respectively. Nuclei are stained with DAPI (blue). In all panels, the genotype is *dlg5*^*EP2087*^*/dlg5*^*EP2087*^*; Ubi-Dlg5.GFP/TM3,Sb*. Scale bars: 20 μm in A and 5 μm in others.

To determine the expression pattern and subcellular localization of Dlg5, we utilized two aforementioned transgenic constructs expressing GFP- or RFP-tagged Dlg5 because we were not able to generate an effective and specific antibody against Dlg5. First, we expressed Dlg5-GFP in the homozygous *dlg5*^*EP2087*^ animals by the *Ubi-Dlg5.EGFP* transgene. The resulting female adults (*dlg5*^*EP2087*^/*dlg5*^*EP2087*^; *Ubi-Dlg5.EGFP*) were viable, fertile and containing follicle epithelia that were completely normal in apical-basal polarity and tissue morphology, indicating that Dlg5-GFP expressed by *Ubi* promoter is able to replace all the function of endogenous Dlg5 in development and follicle epithelial morphogenesis during oogenesis. Therefore, in a strong loss-of-function background (*dlg5*^*EP2087*^/*dlg5*^*EP2087*^), the expression and subcellular localization patterns of Dlg5-GFP are expected to be similar to those of the endogenous Dlg5 protein. We found that Dlg5-GFP displayed two very different subcellular localization patterns in follicle cells of early and late egg chambers respectively (Figs 4A–I” and 5A–E). In the early stage egg chambers (stage 1–8), Dlg5-GFP was localized mainly in the apical membrane and AJ regions of the developing or less mature follicle cells (Figs 4A–E” and 5A–A’”,E, and S3A–D”), consistent with Dlg5’s loss-of-function phenotype. Although Dlg5-GFP mostly overlapped with Crb or aPKC-labeled apical membrane, the Dlg5-GFP signals appeared as a string of connected dots or beads with irregular spacing between any two dots (Figs 4B–D” and 5A–C’”), unlike the continuous line of Crb or aPKC staining (Fig. 4C’’). The *Ubi-Dlg5.EGFP* resulted in Dlg5-GFP expression in both the somatic follicle cells and the germ-line nurse cells, and apical membrane of follicle cells and the apposed membrane of adjacent nurse cells were difficult to be distinguished in early egg chambers. To evaluate contribution of Dlg5-GFP from each of the two membranes, we drove the expression of *UAS-dlg5.RNAi* by *act5C-GAL4,tub-GAL80*^*ts*^ in the *Ubi-Dlg5.EGFP* background to deplete Dlg5-GFP within follicle cells (Fig. S3B–C”). Without Dlg5-GFP expression in follicle cells, Dlg5-GFP localization in the apposed nurse cell membrane was clearly shown to be very mild (Fig. S3B,C). Therefore, most of the Dlg5-GFP signals in the boundary between nurse cells and follicle cells in Fig. S3A were actually localized in the apical membrane of follicle cells. Finally, spots of Dlg5-GFP colocalized very well with strong spots of Arm-labeled AJ (Fig. 4B–B”). And Dlg5-GFP also displayed a moderate level of cytoplasmic staining around lateral region, a portion of which appeared to be at the lateral membrane (Figs 4E–E” and 7F–F”).

However, in the late stage egg chambers (stage 10 and after), Dlg5-GFP was localized mostly to the Dlg-labeled basolateral membrane of the more mature follicle cells, with little signal in the apical membrane and AJ (Figs 4G–I” and 5D–D”’,E). Interestingly, during stage 9 (mid-oogenesis) Dlg5-GFP displayed a localization pattern somewhere in between those of stage 8 and stage 10. Follicle cells surrounding the 15 nurse cells displayed apical and increased basolateral membrane localization, and follicle cells surrounding the oocyte exhibited reduced apical signals and very strong basolateral localization (Figs 4F–F” and 5E). The correlation between Dlg5’s localization pattern change and the dramatic cuboidal-to-columnal cell shape change of follicle epithelium during stage 9 suggests that Dlg5 may somehow play a role in this morphogenetic process. However, we did not have any evidence to support such a hypothesis. Lastly, we sought to determine whether intracellular Dlg5-GFP dots colocalize with β-catenin/Arm in the recycling vesicles as have been previously reported for the mammalian cells. We found no significant colocalization of Dlg5 and Rab11-labeled recycling vesicles, even when significant number of Arm dots colocalized with Rab11 dots (Fig. S4E–E”). Moreover, cytoplasmic Dlg5 spots were found to be partially colocalized with Lva-labeled Golgi but not enriched in Rab5-labeled early endosome and Rab7-labeled late endosome (Fig. S4A–D).

Next, we utilized the genomic construct *Dlg5-TagRFP-T* for analysis of the Dlg5 expression pattern. Since this construct rescued the lethality and epithelial defects caused by Dlg5 loss of function, the 9.6 kb genomic fragment could allow us to better understand the endogenous expression pattern of Dlg5 during development and oogenesis. In the embryos and developing larva, we found that Dlg5-RFP was widely expressed in embryonic epithelia and the imaginal disc epithelia, such as the eye discs, wing discs and leg discs of the 3^rd^ instar larvae (Fig. S5H–K’). Importantly, Dlg5-RFP was broadly expressed throughout different stages of oogenesis, being found in abundance in both the germ-line tissue of nurse cells and oocyte and the somatic follicle cells (Fig. S5A–C). Furthermore, the subcellular localization pattern of the genomic Dlg5-RFP was very similar to that of the *Ubi* expressed Dlg5-GFP, displaying an apical and AJ enrichment in early stage egg chambers and a basolateral membrane localization in late stage egg chambers (Fig. S5D–E”). However, due to a significantly lower level of expression from the genomic construct than from the *Ubi* construct, the fluorescence intensity of Dlg5-RFP is less than that of Dlg5-GFP, resulting in a less sharp localization pattern of Dlg5-RFP when compared to Dlg5-GFP. Taken together, these results indicate that Dlg5 is broadly expressed in a wide variety of developing epithelial tissues and Dlg5’s apical and AJ localization patterns are consistent with its essential roles in apical polarity and AJ maintenance in the developing follicle cells.

### Dlg5 promotes apical membrane localization of Crb

**Figure 6:**
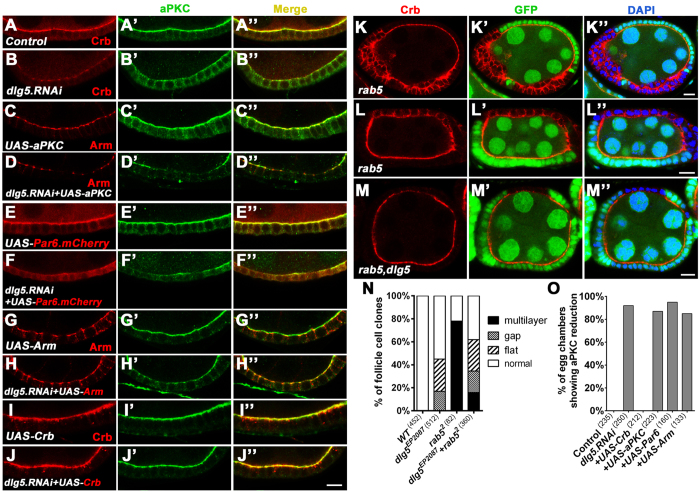
Dlg5 promotes apical localization of Crb. (**A–J”**) Control shows wild-type follicle epithelia (**A–A”**), stained with anti-Crb (red) and anti-aPKC (green). Expression of *UAS-dlg5.RNAi* driven by *act5C-GAL4,tub-GAL80*^*ts*^ caused Crb and aPKC reduction in the apical domain (**B–B”**), as compared to the wild type (**A–A”**). The reduction of aPKC or Crb in apical domain caused by *dlg5* RNAi was not rescued by overexpression of aPKC (**C–D”**), Par6 (**E–F”**) or Arm (**G–H”**), but could be rescued by overexpression of Crb (**I–J”**). In stage 5/6 egg chambers, Crb overexpression caused apical accumulation of Crb and aPKC but not basolateral spread of Crb and aPKC (**I**–**I”**). (**K**–**M”**) In *rab5*^*2*^ mutant clones, Crb either accumulated at cell membrane of cells that lost epithelial organization and became multilayer (**K**–**K”**) or spread to the basolateral domain in cells that still retained epithelial organization (**L**–**L”**). (**M**–**M”**) Both phenotypes were suppressed when the clones are mutant for both *rab5*^*2*^ and *dlg5*^*EP2087*^. Mutant clones were marked by the loss of GFP. (**N**) Quantification of follicle cell morphological defects in wild-type, *dlg5*^*EP2087*^ mutant clones, *rab5*^*2*^ mutant clones and *rab5*^*2*^*,dlg5*^*EP2087*^ double mutant clones. (**O**) Quantification of results of rescue experiment, as shown in (**A**–**J”**). Scale bars: 10 μm.

**Figure 7:**
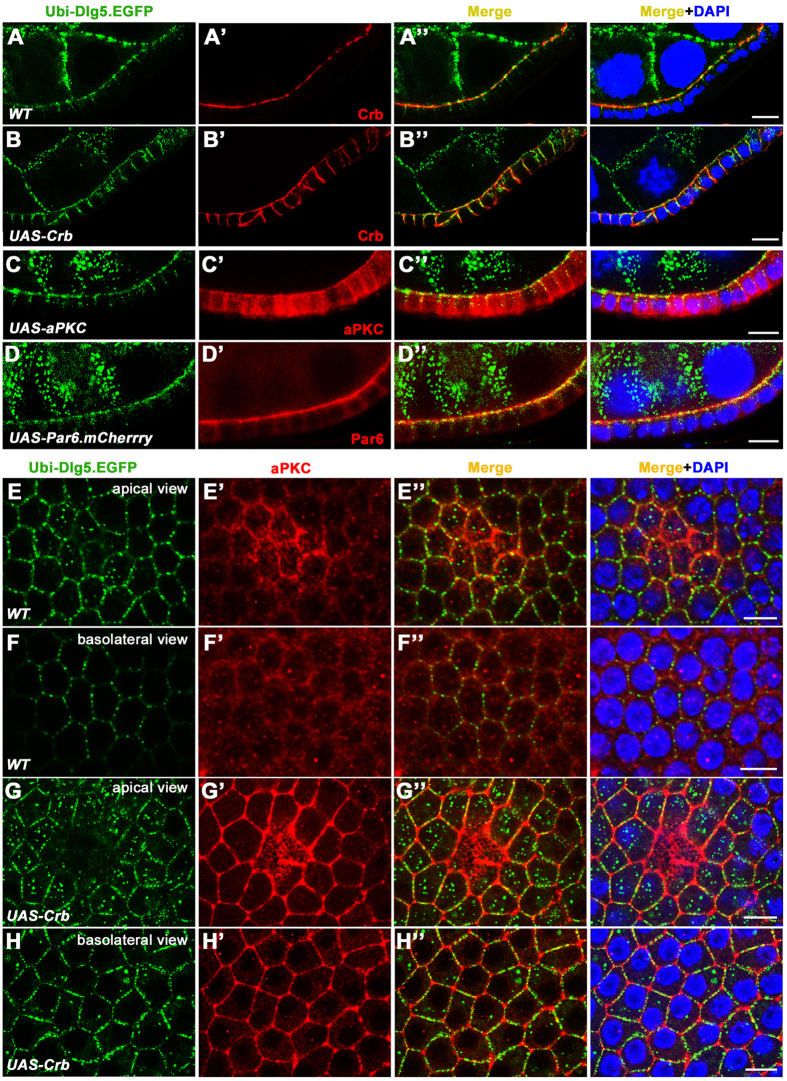
Overexpression of Crb promotes basolateral localization of Dlg5. (**A**–**D”**) In stage 7/8 egg chambers, basolateral spreading of Crb caused by Crb overexpression resulted in increased basolateral localization of Dlg5-GFP (**B**–**B”**), as compared to the wild type (**A**–**A”**). Dlg5-GFP localization was not affected in aPKC or Par6 overexpressing follicle epithelia (**C**–**D”**). (**E**–**H”**) In wild type stage 7/8 egg chambers, single confocal section (planar sectional view) at the apical and basolateral levels showed that Dlg5-GFP was enriched in the apical domain (**E**–**E”**) but not in the basolateral domain (**F**–**F”**). In Crb overexpressing follicle cells, Dlg5-GFP was enriched at both apical (**G**–**G”**) and basolateral membranes (**H**–**H”**). *act5C-GAL4,tub-GAL80*^*ts*^ was used to drive expression in follicle cells. Scale bars: 10 μm.

The immunostaining results demonstrated that distribution of apical polarity markers were most severely affected in *dlg5* mutant follicle cells. It is well known that Crb complex and Par complex function together to define and maintain the apical identity of developing epithelial cells, and disruption in the localization of one complex could lead to failure of apical localization of the other complex^23^. To determine which apical complex was primarily affected by Dlg5 loss of function, we overexpress major components of each complex to perform rescue of the apical polarity phenotype that is resulted from deficiency of Dlg5. Crb, aPKC^24^,^25^, Par6^26^ or Arm^27^,^28^ was each overexpressed in the ovaries expressing *dlg5 RNAi* to determine individual rescue effect (Fig. 6A–J”). As mentioned above, expressing *dlg5 RNAi* in the adult ovary produced similar follicle epithelial defects as those of *dlg5* mutant clones, including strong reduction of apical markers, cell flattening and gaps (Fig. 1E–H, S1A–R’). We found that overexpression of Crb, but not aPKC, Par6 or Arm, was able to fully rescue the reduction of apical markers as caused by *dlg5 RNAi* (Fig. 6A–J”), suggesting that Dlg5 specifically promotes apical localization of Crb. It should be noted that there exits the possibility that aPKC, Par6 or Arm was not being expressed at a high enough level to affect or rescue the *dlg5 RNAi* phenotype. But, it is clear that when comparing to the controls for aPKC and Arm stainings (Figs 4B’ and 6A’,C), overexpression of *UAS-aPKC* (Figs 6C’ and 7C’) or *UAS-Arm* (Fig. 6G) clearly resulted in strong elevation of aPKC or Arm levels. Moreover, Arm overexpression did result in the rescue of reduced Arm staining in AJ, which was caused by *dlg5 RNAi*. When comparing images of *UAS-dlg5 RNAi* or *UAS-dlg5 RNAi* + *UAS-aPKC* (Fig. 6D, S1R’) to those of *UAS-dlg5 RNAi + UAS-Arm* (Fig. 6H), it is apparent that Arm overexpression has a distinct effect on increasing or restoring Arm’s staining levels in AJ and apical domain. Lastly, each of the *UAS-aPKC*, *UAS-Arm* and *UAS-Par6-mCherry* transgenes has been extensively referenced and its overexpression effects documented in the literature^24^,^25^,^26^,^27^,^28^.

Membrane localization of Crb was previously shown to be negatively regulated by endocytosis, and inhibition of Crb endocytosis in *rab5* or *avl* mutant clones resulted in increased membrane localization and ectopic spreading of Crb to the basolateral domain^8^,^29^. If Dlg5 acts to promote membrane localization of Crb, we would expect that deficiency of Dlg5 could suppress the phenotype of membrane accumulation and ectopic spreading of Crb in *rab5* mutant follicle cells. Indeed, *rab5* and *dlg5* double mutations strongly rescued the ectopic spreading of Crb caused by *rab5* single mutation (Fig. 6K–M”). In addition, mutant follicle clones of *rab5* and *dlg5* displayed a similar phenotype to that of *dlg5* mutant clones, which was reduction of Crb in the apical membrane as well as cell flattening and gaps (Fig. 6M–N). Finally, we tested whether ectopic Crb could conversely promote ectopic or increased membrane localization of Dlg5. It was previously known that overexpressing Crb in the follicle cells resulted in the spreading of Crb and consequently aPKC and Par6 to the basolateral membrane of follicle cells during mid-oogenesis^8^,^21^,^30^. We found that ectopic spreading of Crb caused increased membrane localization of Dlg5 in the basolateral membrane (Fig. 7A–B”). On the contrary, overexpression of aPKC or Par6 did not produce such effects (Fig. 7C–D”). Taken together, these results demonstrate that *dlg5* specifically and genetically interacts with *crb* to promote membrane localization of Crb in the apical domain of follicle cells.

### Structure-function analysis

**Figure 8:**
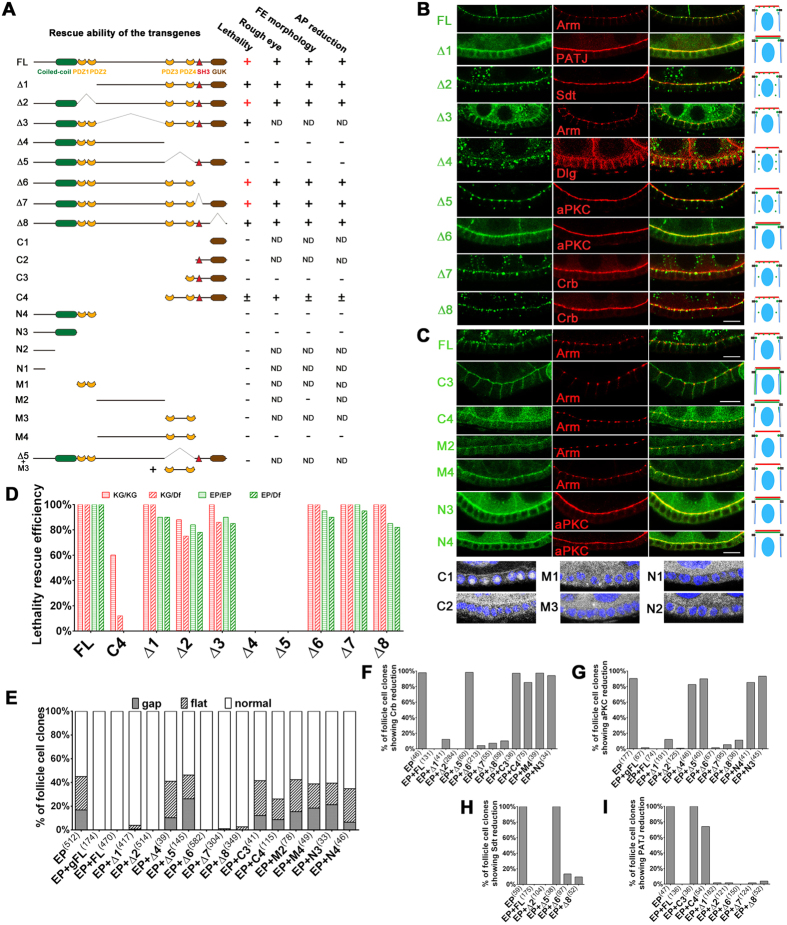
Structure-function analysis of Dlg5. (**A**) Schematics of various truncated forms of Dlg5, transgenes encoding them were used to rescue lethality, rough adult eyes, follicle epithelia’s morphological defects (FE morphology), and apical protein reduction (AP reduction), with the results summarized in the four columns to the right. In the column of lethality rescue, red “+” indicates that the rescued flies can be maintained as stable stocks, “+” indicates moderate rescue effect. ND, not determined. (**B**,**C**) Subcellular localization of different Dlg5 truncated proteins in the follicular epithelia is shown in the first column (green). Co-staining of polarity markers, Arm, PATJ, Sdt, Dlg, aPKC or Crb, is shown in the second column (red). The fourth column shows the schematic diagrams of subcellular localization. The localization of C1, C2, M1, M3, N1, and N2 is shown in the bottom (white). Nuclei are stained with DAPI (blue). Scale bars: 10 μm. (**D**) Quantification of lethality rescue efficiency of various forms of Dlg5 (see Supplementary Table S1 for details). (**E**) Quantification of follicular epithelia morphological defects in different genotypes. (**F**–**I**) Quantification of reduction of apical proteins, including Crb (**F**), aPKC (**G**), Sdt (**H**), PATJ (**I**). KG (*dlg5*^*KG748*^), EP (*dlg5*^*EP2087*^), Df (*Df(2L)BSC242*); gFL (*Dlg5-TagRFP-T* full-length genomic locus), FL (Dlg5 full-length).

As a member of MAGUK superfamily, *Drosophila* Dlg5 contains an N-terminal coiled-coil domain, four PDZ domains, an SH3 domain, and a C-terminal GUK domain (Fig. 1K). To determine which domains or regions are required for Dlg5 function and localization, we generated a large number of *EGFP* or *mRuby* tagged *dlg5* transgenes encoding a spectrum of Dlg5 fragments or truncations that lack certain domains or regions (Fig. 8A). All of the transgenes were expressed under the control of *Ubi-63E* promoter and were inserted in the same attP site on the third chromosome to ensure uniform expression levels from the same genomic loci. We first analyzed the subcellular localization of each truncated protein in the follicle cells, and then determined its abilities to rescue the homozygous lethality, follicle cells’ morphological and apical polarity defects, and the rough appearance of the adult eyes as caused by *dlg5* mutant clones.

Consistent with the aforementioned distribution pattern, the full length Dlg5-GFP (FL), as expressed by *Ubi-Dlg5.EGFP*, was localized to apical domains and AJ of less mature follicle cells in dot-like fashion (Fig. 8B,C FL). The lateral localization of FL at early stage follicle epithelia was weak to moderate. As expected, expression of FL was able to completely rescue the *dlg5* mutant phenotypes, including the lethality, the adult eye large clone defects, the follicle cell morphology phenotype and the reduction of apical complexes (Fig. 8A). Systematically deleting fragments of Dlg5 over the entire length revealed that deletion of the C-terminal region (Δ4, deleting C-terminal PDZ3-PDZ4-SH3-GUK region) produced the most dramatic deviation from FL’s subcellular localization pattern (Fig. 8B Δ4). The Δ4 mutant displayed a cytoplasmic punctate distribution pattern, in contrast to the FL’s membrane localization pattern in apical, AJ and lateral regions (Fig. 8B FL and Δ4). Δ5, a smaller deletion mutant which deletes PDZ3 and PDZ4, retained the strong AJ localization but lost the membrane localization in apical and lateral regions (Fig. 8B Δ5). Moreover, deletions of the N-terminus (Δ1; deleting the N-terminal Coiled-coil-PDZ1-PDZ2 region), part of N-terminus (Δ2; deleting PDZ1-PDZ2 region), Linker region (Δ3), parts of the C-terminus (Δ5, Δ6, Δ7 and Δ8) all resulted in less severe disruptions in the localization patterns than Δ4 (Fig. 8B). Interestingly, Δ4 (deleting PDZ3-PDZ4-SH3-GUK) and Δ5 (deleting PDZ3-PDZ4) are the only deletion mutants that failed to rescue all the *dlg5* mutant defects, whereas all the other deletion mutants were able to fully rescue *dlg5*’s lethality and defects in apical polarity, follicle cell morphology and adult eye appearance (Fig. 8A,D–I and Fig. S6). This result indicates that the C-terminus, and in particular PDZ3-PDZ4, are absolutely essential for Dlg5’s normal functions.

We next determined whether the C-terminal region alone was sufficient for all the Dlg5’s functions. We found that expression of the C-terminal fragment, C4 (including PDZ3-PDZ4-SH3-GUK), was able to partially rescue the lethality of *dlg5*^*KG748*^*/dlg5*^*KG748*^ and *dlg5*^*KG748*^*/Df(2L)BSC242*, but failed to rescue the lethality of *dlg5*^*EP2087*^*/dlg5*^*EP2087*^ and *dlg5*^*EP2087*^*/Df(2L)BSC242* (Fig. 8A,D). Instead of dying at larval stage, 60% of *dlg5*^*KG748*^*/dlg5*^*KG748*^ and 12% of *dlg5*^*KG748*^*/Df(2L)BSC242* animals became viable as adults (Fig. 8D). In addition, C4 fragment displayed a partial rescue effect on cell flattening, gap, and reduction of apical components in the follicle cells as well as a complete rescue effect on the rough phenotype in the adult eyes (Fig. 8A,F,I). On the contrary, expression of the N-terminal fragment (N4), the middle Linker fragment (M2), or any of the smaller fragments (N3, N2, N1, M4, M3, M1, C3, C2, C1) resulted in no significant rescue of the various *dlg5* defects (Fig. 8A,D–G,I). These results indicated that PDZ3 plus the MAGUK core (PDZ4-SH3-GUK) are functionally more important than other regions of Dlg5. We then determined whether any of the large or smaller fragments could be specifically targeted to the apical, AJ, or lateral membrane of the early stage follicle epithelia. We found that the N-terminal N3 (Coiled-coil) and N4 (Coiled-coil-PDZ1-PDZ2) fragments displayed a strong apical membrane localization (Fig. 8C N3 N4), while C3 (MAGUK core) and C4 (PDZ3 plus MAGUK core) both displayed membrane targeting to apical, AJ and basolateral regions, except that C3’s membrane targeting ability is significantly stronger than that of C4 (Fig. 8C C3 C4). Moreover, the middle M2 (Linker) and M4 (Linker-PDZ3-PDZ4) fragments were specifically localized to the Arm-labeled AJs, with M4 displaying a stronger AJ localization than M2 (Fig. 8C M2 M4). Interestingly, the small M3 fragment containing both PDZ3 and PDZ4 exhibited a diffused and cytoplasmic localization pattern lacking any membrane localization (Fig. 8C M3), indicating that PDZ3 and PDZ4 contained no membrane targeting function on their own but they enhanced Linker’s AJ targeting.

## Discussion

Our study demonstrates that Dlg5 is specifically required for the maintenance of apical polarity and AJ of follicle epithelia during early stages of oogenesis, since both loss-of-function mutation and RNAi knockdown of *dlg5* affected only apical polarity regulators and the sub-apical AJ components but not the basolateral regulators. Furthermore, the apical markers (Crb, Sdt, Patj, aPKC and Par6) were more severely reduced than the sub-apical AJ markers (Arm, E-cad and Baz) with the exception of N-cad, suggesting that the loss of apical polarity is the main cause of severe morphological defects in Dlg5-deficient follicle cells. A previous study found that loss of Crb, aPKC and Par6 did not affect the lateral localization of Dlg, whereas loss of Arm caused Dlg spreading to the apical membrane of follicle cells^31^. This is consistent with our result that no apical spreading of Dlg was observed in *dlg5* mutant clones, further confirming that loss of Dlg5 affected apical polarity more severely than the AJ function. Indeed, rescue of apical polarity defects by Crb but not Arm expression further validated this notion.

Importantly, we demonstrate for the first time that Dlg5 positively regulates apical polarity by specifically promoting Crb’s apical membrane localization, based on our following results. First, double staining revealed that Crb reduction was sometimes more severe than reduction of other apical markers (aPKC and Par6; Fig. 3B””) in *dlg5* mutant clones. Second, overexpression of Crb but not other apical or AJ regulators (aPKC, Par6, Arm) could completely rescue *dlg5*’s apical polarity defects. Third, the increased membrane localization and membrane spreading of Crb as caused by blocking the Rab5-mediated endocytosis could be dramatically suppressed by *dlg5* mutation. Moreover, the apical enrichment of Dlg5 in the early and mid-stage follicle epithelia (stage 1-stage 9) further suggests that Dlg5 could function at the apical region to promote Crb’s membrane localization. On the other hand, Crb might conversely enhance Dlg5’s localization to the apical membrane, since overexpression of Crb and hence its membrane spreading toward the basolateral region led to stronger localization of Dlg5 in the basolateral membrane. A previous study has reported that deletion of *Dlg5* in mouse resulted in loss of aPKC but not Par3 (homologous with *Drosophila* Baz) in the developing lung epithelia and that Dlg5 was partially colocalized with aPKC, which are similar to our findings^16^. But how Dlg5 regulated the apical polarity during mouse lung morphogenesis was not understood. Based on our results, it would be worthwhile to check whether murine Dlg5 promotes apical polarity by primarily regulating one of the three mammalian CRB paralogs (CRB1, CRB2, CRB3).

As a MAGUK family member, Dlg5 is thought to function as a scaffold protein. Previous works have focused on which domains of Dlg5 physically interact with junctional and membrane-bound proteins like β-catenin, vinexin and smoothened, and trafficking regulators like syntaxin 4 *in vitro* or in cultured cells^9^,^11^,^15^. But whether such domains are essential for its function and localization *in vivo* and which domains possess apical or AJ membrane targeting ability have not yet been addressed. Our structure-function analysis demonstrates that the C-terminal fragment including MAGUK core (GUK, SH3, PDZ4) and PDZ3 is necessary but not entirely sufficient for Dlg5’s functions. Furthermore, deletion of this C-terminal fragment (Δ4) caused most of Dlg5 to re-distribute to the cytoplasm, losing its membrane localization in the apical, AJ, and lateral regions. Interestingly, PDZ3 and PDZ4 (a subset of C terminal fragment) were also required for Dlg5’s functions, and their deletion (Δ5) likewise resulted in the loss of apical and lateral (but not AJ) membrane localization. One interesting difference between the localization patterns of the other deletion mutants (that still possessed rescue abilities; Δ1–3, Δ6–8) and the patterns of Δ4 and Δ5 is that other deletions all retained some degree of apical localization, in contrast to the lack of localization in the apical membrane for Δ4 and Δ5 (Fig. 8B). Together, these results suggest that MAGUK core and PDZ3’s requirement for Dlg5’s membrane localization in general and PDZ3-PDZ4’s requirement for Dlg5’s apical membrane localization may be critical for Dlg5’s functions in the follicle cells. This is consistent with Dlg5’s role in promoting Crb’s apical localization. Lastly, we found that the N-terminal coiled coil domain, the middle linker region and the MAGUK core could be individually membrane-targeted to apical, AJ and all (apical, AJ and basolateral) regions respectively.

## Materials and Methods

### Drosophila genetics

Flies were cultured following standard procedures at 25 °C except for RNAi experiments at 29 °C. All strains were obtained from the Bloomington *Drosophila* Stock Center, except for the following: *dlg5*^*KG748*^*, FRT40A/CyO*^19^, *dlg5*^*EP2087*^*/CyO* (Szeged stock Center), *Rab5*^2^*, FRT40A/CyO*^32^, *Ecad-GFP*^33^, *Par6-GFP*^34^, *UAS-Par6.mCherry*^26^, *UAS-aPKC*^24^, *Nrg-GFP*^35^. The *UAS-dlg5.RNAi* strains employed were: GD22496, GD46234 and KK101596 from Vienna *Drosophila* RNAi Center (only the first one was shown), 30925 from Bloomington *Drosophila* stock center. The *dlg5*^*KG748*^ mutant was backcrossed to *w1118* for 15 generations to outcross the background mutations. Clonal analyses in the adult ovaries were performed using the hs-Flp/FRT systems with GFP or RFP as marker for wild-type cells. Flies were dissected one day after clone induction by heat shock. To generate large clone mosaic eyes, the ey-Flp/FRT system combined with *Minute* mutation was used. Experiments with the temperature sensitive Gal80^ts^ system were carried out at 18 °C to repress GAL4-mediated transcriptional activation, which was induced at 29 °C.

### Transgenes

To generate the genomic construct, 9.6kb genomic sequence of *dlg5* locus was amplified from the BAC CH321-38E02 (Drosophila Genomics Resource Center (DGRC)) and subcloned into the insulated pattB vector (pattB2G, modified from the original pattB vector^36^). The TagRFP-T fluorescent tag was inserted in-frame in the upstream of the stop codon, resulting the *Dlg5-TagRFP-T* construct (Fig. 1L). To generate the ubiquitous expression construct *Ubi-Dlg5.3XFlag*, Dlg5 CDS was amplified from LD32687 (DGRC) and subcloned into the vector pUbFSTF.attB. To generate the RNAi rescue construct *UAS-Dlg5*^*RNAiRescue*^, the N-terminal and C-terminal encoding segments of *Drosophila melanogaster dlg5* (LD32687; DGRC) and the largest exon of *Drosophila pseudoobscura dlg5* (CH1226-65H17; DGRC) were PCR amplified, ligated in frame, and subcloned into the insulated pUASTattB vector. The *dlg5* constructs for structure-function analysis, including the full-length construct, were produced by PCR amplification of specific *dlg5* regions from LD32687 (DGRC). Fragments were ligated and inserted into the pUbGFP.attB or pUbRuby.attB vectors, which contain a Ubiquitin-63E promoter and a C-terminal GFP or mRuby tag^37^. The deletion constructs encode the following amino acid sequence segments of Dlg5: FL, 1-1916; Δ1, 611-1916; Δ2, 1-448 and 611-1916; Δ3, 1-620 and 1289-1916; Δ4, 1-1296; Δ5, 1-1296 and 1580-1916; Δ6, 1-1591; Δ7, 1-1592 and 1659-1916; Δ8, 1-1735 and 1905-1916; C1, 1659-1916; C2, 1580-1916; C3, 1472-1916; C4, 1289-1916; M1, 430-620; M2, 611-1296; M3, 1289-1591; M4, 609-1584; N1, 1-127; N2, 1-217; N3, 1-448; N4, 1-620. All the constructs were sequenced and inserted into the attP2 docking site except the genomic *Dlg5-TagRFP-T* construct which was inserted into the attP4 site. The P[acman] BAC CH322-120K05 from DGRC was inserted into the 86Fa attP site. All the transformations are performed using established PhiC31-based methods.

### Immunohistochemistry and microscopy

Ovary dissection was carried out in phosphate-buffered saline (PBS) and then fixed in Devitellinizing buffer (7% formaldehyde) and heptane (Sigma) mixture (1:6) for 10min. After washes in PBS, ovaries were incubated in blocking solution (PBT, 10% goat serum) for 30min and then stained overnight at 4 °C. Primary antibodies and their concentrations were as follows: mouse anti-Arm (1:50; N2 7A1; Developmental Studies Hybridoma Bank (DSHB)), rabbit anti-PKCζ (1:200; C-20; Santa Cruz), mouse anti-Crb (1:10; Cq4; DSHB), rat anti-Ncad (1:20; DN-Ex #8; DSHB), mouse anti-β-tubulin (1:500; E7; DSHB), rabbit anti-Baz (1:400; kind gift from A. Wodarz), mouse anti-Dlg (1:50; 4F3; DSHB), rabbit anti-Sdt (1:500)^38^, rabbit anti-PATJ (1:1000)^39^, rabbit anti-Lva (1:200)^40^, rabbit anti-Rab5 (1:100, ab31261, Abcam), rabbit anti-Rab7 (1:3000)^41^ and rabbit anti-Rab11 (1:8000)^41^. Methanol treatment was used after fixation for anti-Crb staining. After washes in PBT, ovaries were incubated with secondary antibodies (1:250, Jackson ImmunoResearch) for 2 hours at room temperature. F-actin was labeled by Rhodamine phalloidin (1:100, Sigma). Confocal images were obtained using a Leica TCS SP5 II or an Olympus FV1000 confocal microscope.

### RNA extraction and Real-time PCR

*w1118*, *dlg5*^*KG748*^*/CyO,GFP*, *dlg5*^*EP2087*^*/CyO,GFP* flies were kept in cages and GFP-negative larvae were collected at 2^nd^ instar larval stage. Total RNA was isolated using the SV Total RNA Isolation System (Promega). 1 μg of total RNA was used for cDNA synthesis using the SuperScript II reverse transcriptase kit (Invitrogen). PCR was performed in a total volume of 20 μl following the manufacturer’s instructions for the SYBR Green assay (Applied Biosystems). Reactions were run in triplicate on a StepOnePlus Real-Time PCR System (Applied Biosystems). Before each experiment, the calibration curves were validated. Samples whose curves amplified out of the calibrated dynamic range were eliminated. All results were normalized to the *rp49* mRNA level and calculated using the ΔΔCt method. Statistical significance was determined by two-tailed, unpaired Student’s *t* test in GraphPad Prism software.

## Additional Information

**How to cite this article**: Luo, J. *et al.* Dlg5 maintains apical polarity by promoting membrane localization of Crumbs during *Drosophila* oogenesis. *Sci. Rep.* **6**, 26553; doi: 10.1038/srep26553 (2016).

## Supplementary Material

Supplementary Information

## Figures and Tables

**Figure 5:**
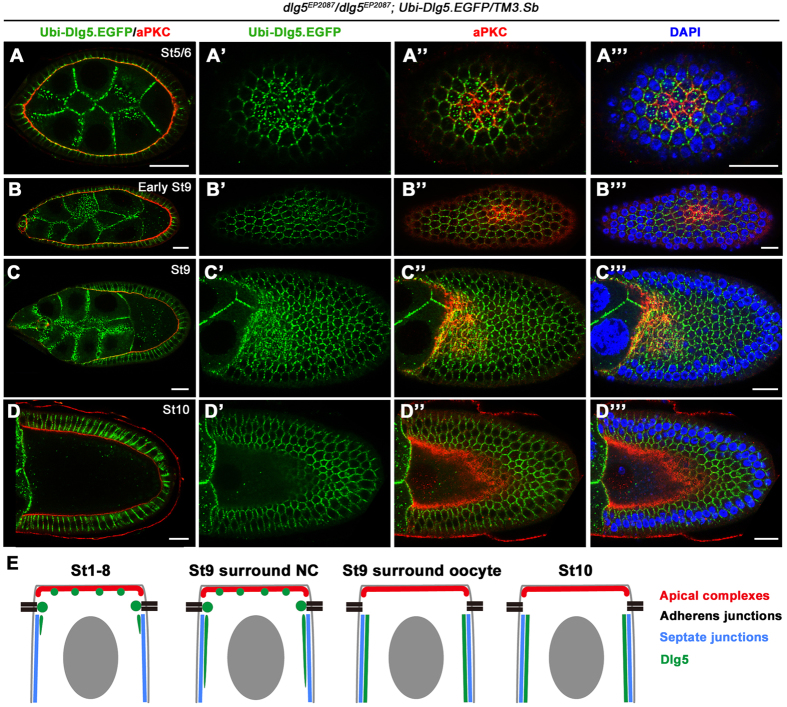
The spot-like localization of Dlg5 is restricted in apical domain of early and mid-stage egg chambers. The left column represents the transverse sectional views and the other columns represent the planar sectional views of the same egg chambers. (**A–C”’**) In early stage and stage 9 egg chambers, Dlg5-GFP showed punctate localization pattern in apical domain marked by aPKC. Note that due to the curvature of egg chambers, a single planar section views both apical domains (including marginal zone, marked by aPKC) in the center and the surrounding sub-apical AJs. (**D–D”’**) In stage 10 egg chamber, Dlg5-GFP localized to the basolateral domain and had no spot-like localization. (**E**) Schematic diagram for Dlg5 localization in follicle cells at different stage egg chambers. Scale bars: 20 μm.
