# Supplementary material for: Dlg5 maintains apical polarity by promoting membrane localization of Crumbs during Drosophila oogenesis
Source: Sci Rep. 2016 May 23;6:26553. doi: 10.1038/srep26553 (PMC4876392; doi:10.1038/srep26553)
Supplement: Supplementary Information [file srep26553-s1.pdf]

Supplementary information

Dlg5 maintains apical polarity by promoting membrane localization of Crumbs during *Drosophila* oogenesis

Jun Luo[1], Heng Wang[1], Di Kang[1], Xuan Guo[1], Ping Wan[1], Dou Wang[1], Jiong Chen[1]*

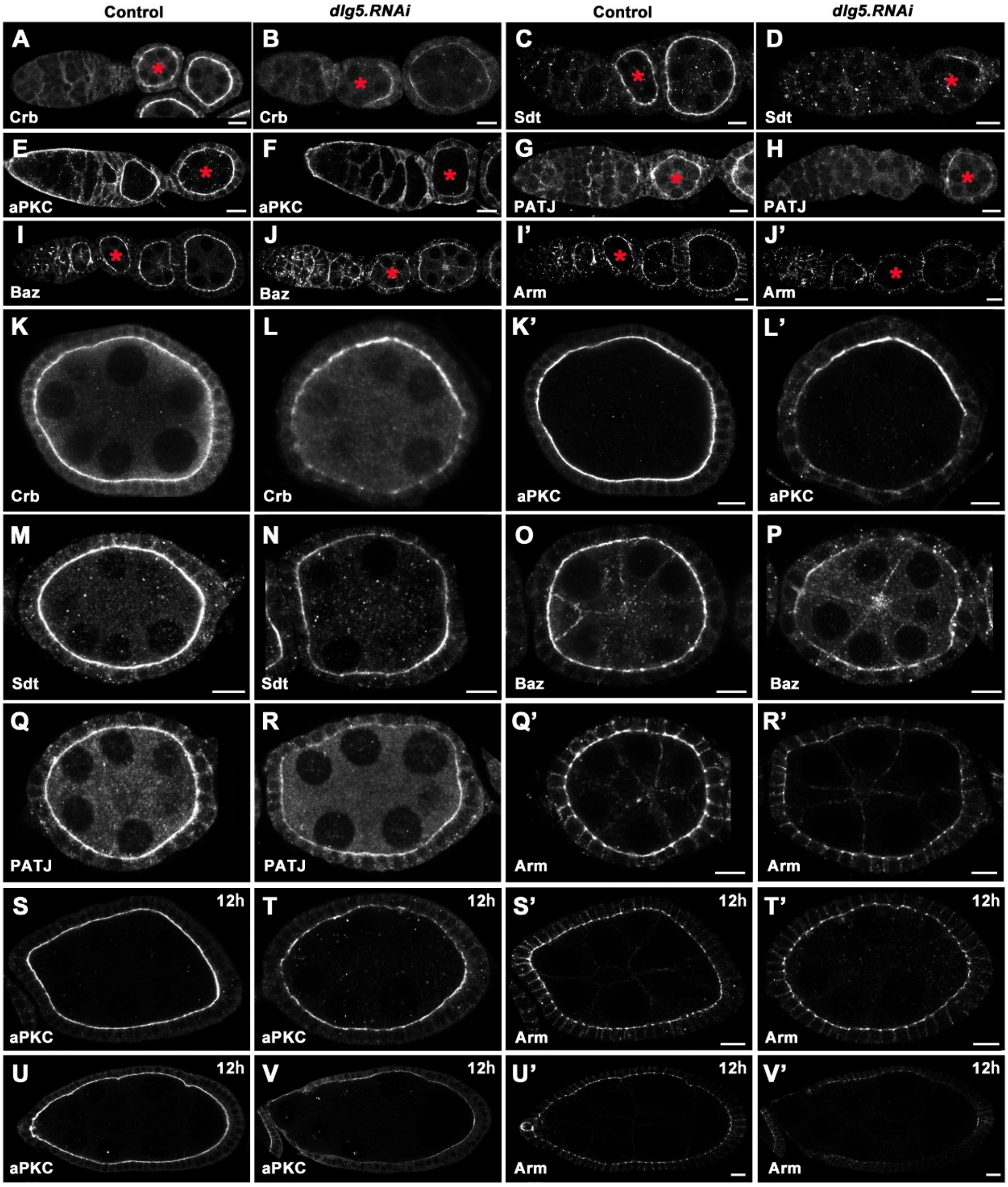

Figure S1. RNAi knockdown of *dlg5* confirms that Dlg5 is required for maintenance of apical polarity. *dlg5* RNAi was driven by *act5C-GAL4,tub-GAL80^{ts}* system and induced at 29°C for 3 days (A-R'), or for 12h (S-V'). The first and third columns display the control egg chambers stained with different markers, and the second and fourth column display *dlg5* RNAi egg chambers stained with different markers. (A-J') In *dlg5* RNAi ovaries, apical and AJ proteins including Crb (A,B), Sdt (C,D), aPKC (E,F), PATJ (G,H), Baz (I,J) and Arm (I',J') were clearly reduced in stage 1 egg chambers (indicated by red asterisks). (K-R') In other older *dlg5* RNAi egg chambers, Crb (K,L), aPKC (K',L'), Sdt (M,N), Baz (O,P), PATJ (Q,R) and Arm (Q',R') were also reduced. (S-V') After only 12 hours of *dlg5* RNAi, significant aPKC (T,V) and Arm (T',V') reductions were observed in stage 6/7 (S-T') and early stage 9 egg chambers (U-V'). Scale bars: 10µm.

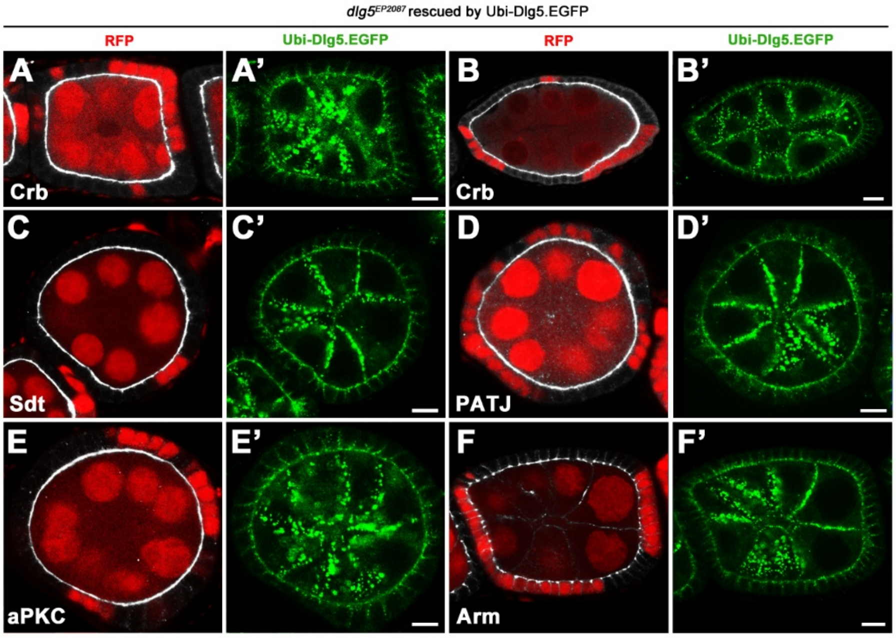

Figure S2. The apical polarity and AJ defects in *dlg5* mutant clones are rescued by *Ubi-Dlg5.EGFP*. (A-F') The reductions of apical and AJ proteins in *dlg5^{EP2087}* clones were completely rescued by *Ubi-Dlg5.EGFP* (green). The apical proteins including Crb (A-B'), Sdt (C-C'), PATJ (D-D'), and aPKC (E-E') are shown in white. The AJ component Arm (F-F') is shown in white. Mutant clones were marked by the loss of RFP. Scale bars: 10µm.

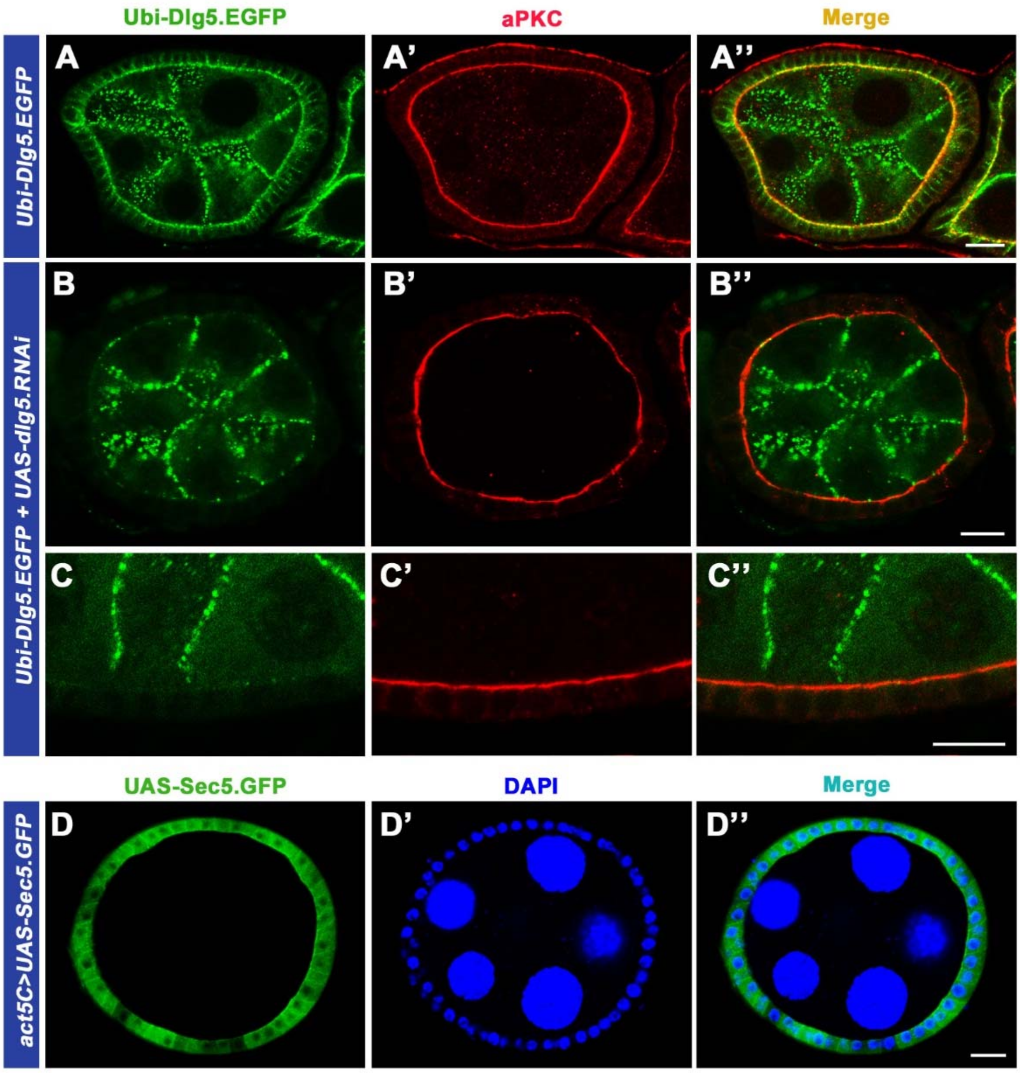

Figure S3. *dlg5 RNAi* knockdown driven by *act5C-GAL4* reveals most of Dlg5-GFP signals are in the apical domain of follicle cells but not in the apposed nurse cell membrane. (A-A'') *Ubi-Dlg5.EGFP* allowed Dlg5-GFP to be expressed in both the somatic follicle cells and the germ-line nurse cells. Dlg5-GFP colocalized with aPKC in the follicle cell's apical membrane, which is difficult to be distinguished from the juxtaposed nurse cell membrane. (B-C'') *UAS-dlg5.RNAi* driven by *act5C-GAL4,tub-GAL80^{ts}* in the *Ubi-Dlg5.EGFP* background resulted in almost complete depletion of Dlg5-GFP specifically in the follicle cells, but Dlg5-GFP distribution in the nurse cell membrane and cytoplasm was not significantly affected. This result indicates that Dlg5-GFP localization in the nurse cell membrane (juxtaposing follicle cell's apical surface) was mild and most of the Dlg5-GFP signals in the boundary between nurse cells and follicle cells were actually localized in the apical region of follicle cells. Note that image

in (C-C”) was shown at a higher magnification than the image in (B-B”). (D-D”) Expression of *UAS-Sec5.GFP* that is driven by *act5C-GAL4* resulted in the exclusive distribution of Sec5-GFP (a cytoplasmic exocyst component protein) in the follicle cells, indicating that *UAS-transgene* expression driven by *act5C-GAL4,tub-GAL80*^{ts} is restricted only to follicles cells and not in the nurse cells. Note that no GFP signal was detected in the nurse cells. Scale bars: 10μm.

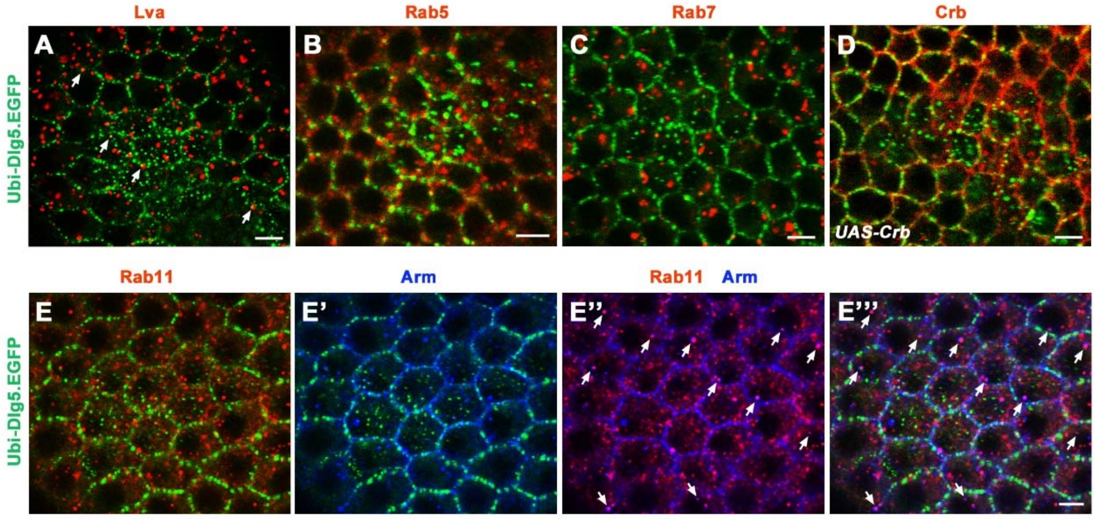

Figure S4. Dlg5 appeared not to colocalize to Crb or Arm containing vesicles. (A-E’’’) In *Ubi-Dlg5.EGFP* expressing follicle cells, most of cytoplasmic Dlg5-GFP spots were not colocalized with early endosome (marked by Rab5; B), late endosome (marked by Rab7; C), or recycling endosome (marked by Rab11; E), except for Golgi (marked by Lva; A), where a few spots of Dlg5-GFP were seen localized with Lva (pointed by white arrows). Cytoplasmic Arm spots were observed to partially colocalize with Rab11-labeled recycling endosome (pointed by white arrows in E”), but they did not colocalize with cytoplasmic Dlg5-GFP spots (E’, E’’’). Scale bars: 10μm.

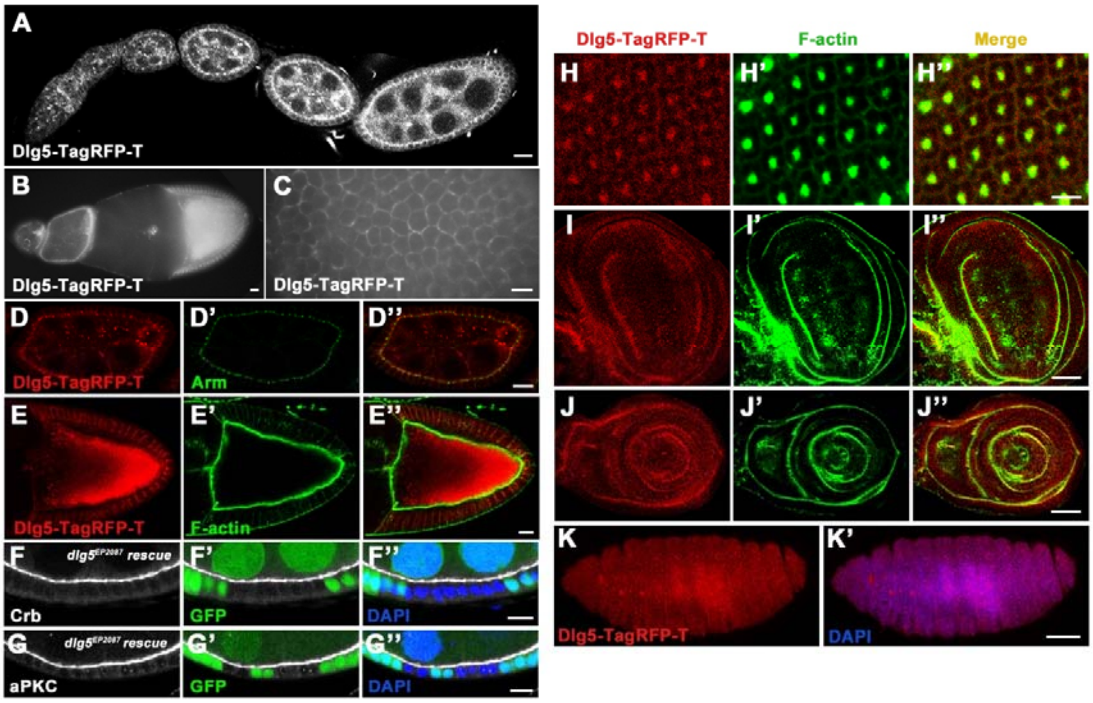

Figure S5. Dlg5 is widely expressed in embryonic, imaginal and follicular epithelia. (A-E'') The genomic construct *Dlg5-TagRFP-T* indicates that Dlg5 (white) is broadly expressed throughout different stage of oogenesis (A-C). Dlg5 (red) localized to the apical domain and AJs in early stage egg chambers (D-D'') and to the basolateral domain in late stage egg chambers (E-E''). (F-G'') Follicle cell morphological defects and reduction of apical proteins were rescued by the genomic construct *Dlg5-TagRFP-T*. The apical proteins Crb (F-F'') and aPKC (G-G'') are shown in white. Mutant clones were marked by the absence of GFP (green). Nuclei were stained with DAPI (blue). (H-J'') Dlg5-TagRFP-T (red) was widely expressed in the imaginal disc epithelia of 3rd instar larvae, such as the eye disc (H-H''), the wing disc (I-I'') and the leg disc (J-J''), as well as in the embryonic epithelium (K,K'). Scale bars: 10μm in A-H' and 50μm in I-K'.

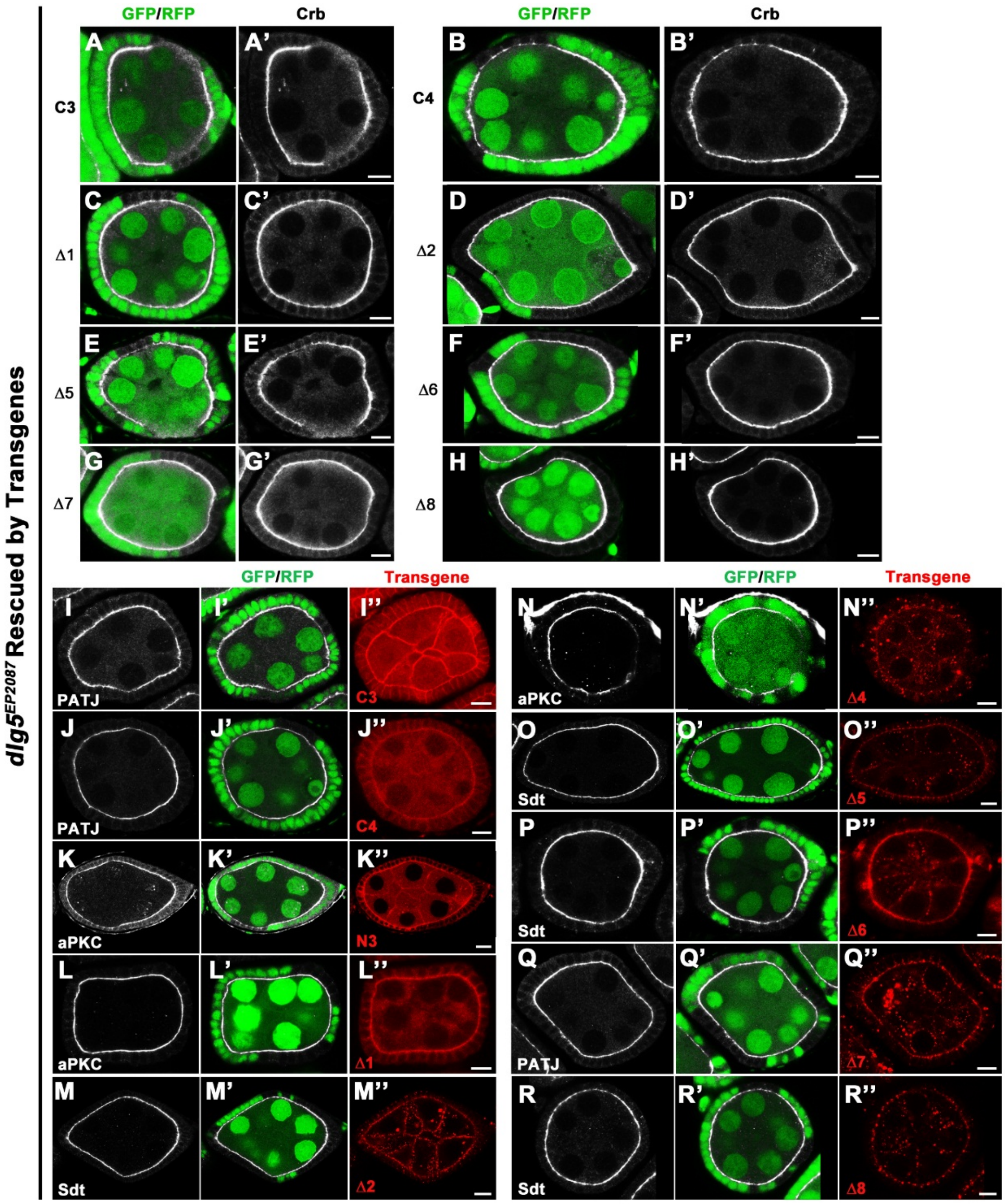

Figure S6. Ability of various truncated forms of Dlg5 to rescue the apical reduction of Crb, aPKC, Sdt and PATJ in *dlg5^{EP2087}* mutant clones. (A-R'') Confocal images showing different Dlg5 truncation forms rescuing Crb, aPKC, Sdt or PATJ reduction in *dlg5^{EP2087}* mutant clones. Δ1, Δ2, Δ6, Δ7 and Δ8 strongly rescued the apical proteins reduction (C-D',F-H',L-M'',P-R''); C4 partially rescued the apical proteins reduction (B-B',J-J''); whereas C3, N3, Δ4 and Δ5 had no rescue ability (A-A',E-E',I-I'',N-N'',O-O''). Mutant clones were marked by the absence of the GFP or RFP (green). Scale bars: 10μm.

Table S1. Lethality rescue efficiency of Dlg5 transgenes.

Transgenes	$dlg5^{KG748}$	$dlg5^{EP2087}$	$dlg5^{KG748}/Df^{*}$	$dlg5^{EP2087}/Df^{*}$
CH322-120K05	93% (n=185)	88% (n=232)	NA	NA
Dlg5-TagRFP-T	100% (n=1119)	90% (n=701)	100% (n=424)	88% (n=575)
Ubi-Dlg5.3XFlag	100% (n=396)	100% (n=334)	100% (n=380)	100% (n=542)
Ubi-Dlg5.EGFP	100% (n=358)	100% (n=733)	100% (n=323)	100% (n=282)
C4	60% (n=198)	12% (n=245)	0% (n=285)	0% (n=398)
Δ1	100% (n=283)	100% (n=376)	90% (n=214)	90% (n=253)
Δ2	88% (n=297)	75% (n=268)	84% (n=350)	78% (n=280)
Δ3	100% (n=253)	86% (n=389)	90% (n=275)	85% (n=268)
Δ4	0% (n=328)	0% (n=354)	0% (n=326)	0% (n=488)
Δ5	0% (n=386)	0% (n=392)	0% (n=454)	0% (n=390)
Δ6	100% (n=186)	100% (n=372)	95% (n=409)	90% (n=237)
Δ7	100% (n=248)	100% (n=381)	100% (n=296)	95% (n=248)
Δ8	100% (n=182)	100% (n=255)	85% (n=282)	82% (n=248)

* *Df*, *Df(2L)BSC242*
